# Effect of an Outdoor Access System on the Growth Performance, Carcass Characteristics, and *Longissimus lumborum* Muscle Meat Quality of the Prestice Black-Pied Pig Breed

**DOI:** 10.3390/ani10081244

**Published:** 2020-07-22

**Authors:** Anne Dostálová, Alena Svitáková, Daniel Bureš, Libor Vališ, Zdeněk Volek

**Affiliations:** 1 Departments of Nutritional Physiology and Animal Product Quality, Institute of Animal Science, Přátelství 815, 104 00 Prague Uhříněves, Czech Republic; volek.zdenek@vuzv.cz; 2 Departments of Genetics and Breeding of Farm Animals, Institute of Animal Science, Přátelství 815, 104 00 Prague Uhříněves, Czech Republic; svitakova.alena@vuzv.cz; 3 Department of Cattle Breeding, Institute of Animal Science, Přátelství 815, 104 00 Prague Uhříněves, Czech Republic; bures.daniel@vuzv.cz; 4 Department of Pig Breeding, Institute of Animal Science, Přátelství 815, 104 00 Prague Uhříněves, Czech Republic; libor.valis@mze.cz

**Keywords:** carcass composition, feed efficiency, indigenous pig breed, nutrition, pasture, sensory analysis

## Abstract

**Simple Summary:**

The conservation of indigenous animal breeds is important, in terms of global food security and agricultural sustainability. The Prestice Black-Pied pig is a Czech breed which is maintained under the National Program for the Conservation and Utilization of Genetic Resources. Compared to improved breeds, the Prestice pig is characterized by a lower growth performance and higher carcass fatness; and is therefore not competitive under large-scale rearing conditions. On the other hand, so-called “primitive” characteristics, such as hardiness and adaptability, have been preserved in this breed. Thus, they are suitable for extensive rearing conditions similar to other indigenous European breeds. Despite this, most of the Prestice pig population is kept within a conventional indoor system. No research has been performed on the performance of this genotype within extensive conditions and their subsequent meat quality. Based on the results of the present study, the Prestice pig is able to utilize local protein feed (white lupin seeds/pea seeds) well, with regards to growth and feed efficiency, and it is possible to fatten them extensively using an outdoor-access system. An in-depth assessment of the breed’s production potential and market potential could assist in the conservation of the breed; through the promotion of higher value products.

**Abstract:**

The effect of an outdoor-access vs. conventional indoor system on the growth, carcass characteristics, and *longissimus lumborum* muscle (LL) meat quality was evaluated in 24 Prestice Black-Pied pigs, during the growing-finishing period. Two groups received the same complete diet and were housed separately under conventional indoor conditions, with only one group having full access to pasture (350 m^2^/pig). The animals showed acceptable growth rates (outdoor vs. indoor, average of 740 g/d vs. 700 g/d), feed intake (average of 2700 g/d), and feed conversion ratios (FCR) (average of 3.3 vs. 3.5). The rearing system significantly affected the fatty acid composition of the LL. Outdoor pigs had lower ratios of n − 6/n − 3 polyunsaturated fatty acids, saturation indexes, atherogenic indexes, and thrombogenic indexes, compared with indoor-raised pigs. No differences were recorded in carcass characteristics, physical meat quality traits (pH_45_, pH_24_, drip loss, water holding capacity), or the chemical composition of the meat (crude protein, cholesterol, intramuscular fat, hydroxyproline, and tocopherol). The sensory analysis of grilled LL muscle found that outdoor pigs received lower evaluation scores for tenderness, juiciness, and chewiness, but had a better overall acceptance compared to pigs reared indoors.

## 1. Introduction

The Prestice Black-Pied pig (PBP) is an indigenous Czech breed which has been reared in a closed population within the National Program on the Conservation and Utilization of Genetic Resources since 1992 [1]. This breed is characterized by strong conformation, good maternal qualities, adaptability, longevity, and resistance to diseases. Compared with modern breeds selected for high production performance, the PBP has slower growth rates, lower lean meat percentages, and higher carcass fatness [2]. Similar to other indigenous European pig breeds, the PBP has a higher amount of intramuscular fat, which is typically beneficial for meat tenderness and juiciness [2,3]. Based on the abovementioned characteristics, the PBP should be considered for fattening in extensive systems, similar to other European local breeds such as the Wessex Saddleback, Cinta Senese, and Iberian pigs. Rearing in an outdoor system is considered to be a more favorable environment for animals in terms of welfare, because a number of natural behaviors can be more easily demonstrated [4], and pathogenic infection rates can be decreased [5]. From a human nutrition perspective, outdoor systems improve some nutritional characteristics of pork products, such as the fatty acid profile, in favor of polyunsaturated fatty acids and n3 PUFA [6], which are beneficial for human health [7]. Moreover, a higher content of tocopherol has been found in products from free-range systems with grazing access [6], which is desirable due to its antioxidant characteristics, preventing lipid and protein oxidation [8,9], and thereby potentially improving the shelf life of meat products [10,11]. Additionally, other meat characteristics, such as eating quality, have been ascribed to interactions between genetic and environmental factors [12].

Currently, most of the PBP population is kept in conventional indoor systems, and currently no data have been recorded regarding raising the PBP in an outdoor-access system, which seems to be more suitable for maintaining this unique and limited genetic resource. Although outdoor/free-range pork production represents a small share in the current market, it appears to meet the increasing consumer demand for sustainable and ethical meat products. This study thus describes the effect of utilizing an outdoor-access system on the growth, carcass characteristics, and *longissimus lumborum* muscle meat quality of the PBP, while using an experimental diet based on locally-produced protein sources (white lupin seeds and pea seeds). These results may be applicable for breeders interested in the utilization of a low-input system and wanting to produce special-label products (“free-range/high welfare”).

## 2. Materials and Methods

The experiment was conducted at the experimental farm of the Institute of Animal Science (IAS) in Prague (50.0308042° N, 14.6053619° E) [13], following EU directive [13]. All the experiment procedures were approved by the Animal Care Committee of IAS.

### 2.1. Animals and Experimental Design

A total of 24 two-month-old Prestice Black-Pied pigs (12 barrows and 12 gilts) with an average body weight of 22 ± 6 kg were randomly divided into two groups, ensuring equal sex ratios. The animals originated from the same breeding farm, but from different litters, and were weaned after 28 days from birth. Both groups—the outdoor group (OG) and indoor group (IG)—were loose-housed in two pens (1 m^2^/pig) with straw bedding, within the IAS experimental stable. The experiment was conducted during the late spring and summer seasons, with mean temperatures being between 15 and 32 °C (May–September).

During the trial, the OG had unlimited access to pasture 24 h per day. The pasture area (with a stocking density of 350 m^2^/pig) was divided into three paddocks with different pasture crops (paddock 1: winter wheat, vetch, and rye grass; paddock 2: oat and vetch and rye grass; paddock 3: rye grass). The paddocks were gradually made available, to ensure grazing throughout the experiment. Representative samples of the forage were taken from each paddock before they were opened, and they were used for the determination of the chemical composition of the forage (Table 1). The minimum stocking density on the pasture was calculated to produce a yearly nitrogen (N) deposition of 170 kg N/ha, based on European standards. The first paddock was opened after eight days of adaptation, following animal regrouping and the provision of the total mixed ration. After the pasture was completely utilized in the first paddock, the second was opened to the OG, and subsequently the third. Both groups were fed ad libitum with the same total mixed ration throughout the experiment. The experimental diet was formulated with regards to the PCBs fattening abilities, and using locally-produced, non-GMO protein sources (white lupin seeds and pea seeds), which more accurately reflects the current European regulations and consumer expectations regarding sustainable production (Table 2). The pigs had ad libitum access to water throughout the study.

### 2.2. Feed Chemical Composition

The chemical analysis of the diets followed the official methods of AOAC International [14,15]. Dry matter was analyzed according AOAC 991.02, during which diet samples were dried at 103 ± 2 °C using a Memmert dryer (ULM600, Memmert, Swabach, Germany). The ash content (as a % of the wet weight) was determined in duplicate, by incineration at 500 °C for 6 h, following AOAC 942.05. Crude protein, crude fiber, and ether extract were determined using a Kjeltec AUTO 1030 Analyzer, Fibertec 2010, and Soxtec 1043 (FOSS Tecator AB, Höganäs, Sweden), respectively, according to methodology described in AOAC 954.61 (crude protein), AOAC 978.10 (crude fiber), and AOAC 2003.5 (ether extract). The amino acid content of the experimental diet was analyzed after hydrolysis in 6 M hydrochloric acid at 110 °C for 23 h, using an Amino Acid Analyzer AAA-400 (INGOS Ltd., Prague, Czech Republic) equipped with an ion-exchange column. Methionine was analyzed after oxidation with performic acid at 0 °C for 1 h, as cysteic acid and methionine sulfone. A post-column derivation with ninhydrin was used.

### 2.3. Growth Performance

The feeding trial started at the age of two months and finished when the pigs reached a body weight of 110 ± 8 kg, at an age of six months. The animals were weighed three times during the experimental period: at the beginning (end of May), at the end of August, and on the day of slaughter. Feed intake of the total mixed ration was measured every day, on a per group basis. Weight gain and feed conversion ratio were calculated at the end of the trial. Daily weight gain was calculated from the initial and final slaughter weights of the pigs. The feed conversion ratio was calculated using the group consumption of the feeding mixture.

### 2.4. Slaughter and Carcass Composition and Muscle Sampling

All pigs (24) were slaughtered at the same experimental slaughterhouse, belonging to IAS, in September 2014. Feed was withdrawn for 12 h before the animals were transported to slaughter. They were slaughtered by electrical stunning (350 V, 4 A) and exsanguination. Within 45 min after slaughter, the carcasses were dressed and assessed by a qualified classifier for their estimated lean-meat content, according to the ZP Method (Zwei Punkten Methode) [16]. Carcass measurements were performed after 24 h of chilling (at approximately + 2 °C), on the right side of the carcasses. Firstly, backfat thickness (with the skin) was measured at three locations (in the middle of the second *thoracic vertebra*, at the last *thoracic vertebra*, and at the first *sacral vertebra*), and the arithmetic mean was calculated from these measurements. Then, the right carcass sides were weighed and divided into standardized commercial cuts, according to the commercially-used methodology in the Czech Republic [17]. Major carcass cuts, (ham, loin, shoulder, and belly) with bones, were weighed. After dissection, 500 g samples of the *longissimus lumborum* muscle (LL) were cut between 12th and 13th ribs, as counted from the caudal side, and were transported in a cooling box to the laboratory for further analysis.

### 2.5. Physical and Chemical Analysis

The muscle pH was measured twice, at 45 min (pH_45_) and 24 h (pH_24_) after slaughter, using an InoLab pH 730 set (WTW, Weilheim, Germany) equipped with a SenTix Sp probe. A 20 mm-thick steak was cut from the LL muscle of each animal and used for the determination of drip loss as follows: the initial weight of the steak was recorded, and then each sample was suspended inside an inflated polyethylene bag, and stored in a refrigerator at 4 °C for 24 h. The steaks were thereafter removed from the bags, blotted dry with absorbent paper, and individually weighed again. Drip loss was calculated as a percentage of the initial sample weight, as described by Honikel [18]. The water-holding capacity (WHC) was measured using 80 g of homogenized lean muscle, to which 120 mL of distilled water and 5 g of sodium chloride were added, and subsequently mixed together. The homogenate was transferred to a test tube of known weight, and the weight of both the tube and sample was recorded. Subsequently, the tubes were heated in a water-bath at 75 °C for 30 min. The liquid fraction was then disposed of, and the tube with the remaining sample was left in an inverted position for 30 min, where after they were weighed again. The percentage of retained water was determined as follows: 250 × (b − 0.4 × a)/a, where a = weight of the raw homogenate and b = weight of the homogenate after heating and cooling. Thirty hours after slaughter, further LL muscle samples were homogenized and used for chemical analysis. The dry matter content was determined by oven-drying at 105 °C to a constant weight, where after samples were homogenized using a Grindomix GM 200 knife mill (Retsch, Haan, Germany) for crude protein determination. The lean muscle protein content was determined using a Kjeltec 2400 (FOSS Tecator AB, Höganäs, Sweden). The lipid fraction was isolated by extraction with petroleum in a Soctec Avanti 2055 apparatus (FOSS Tecator AB, Höganäs, Sweden), as described in ISO 1444 [19]. Hydroxyproline content in dried, de-fatted muscle samples was determined with a spectrophotometer (Varian Cary 50 Probe, Mulgrave, Australia), according to the methodology described by Bergman and Loxley [20]. The cholesterol content was determined by lipid extraction with diethyl ether, after saponification, and in accordance with ISO 3596 [21]. The fatty acid (FA) composition of the *longissimus lumborum* muscle was assessed after chloroform-methanol extraction of total lipids, according to Folch [22], and as previously described by Volek [23]. Briefly, after alkaline trans-methylation of FA ISO 5509 [24], gas chromatography was performed on these extracted samples with a HP 6890 gas chromatograph (Agilent Technologies, Inc., Santa Clara, CA, USA) according to ISO 5508 [25]. The gas chromatograph was equipped with a DB-23 cyanopropyl-methylpolysiloxane column (150 to 230 °C). Fatty acids were identified on the basis of retention times corresponding to the standards used (PUFA 1, PUFA 2, PUFA 3, CRM-Supelco 37 Component FAME Mix; Supelco, Bellefonte, PA, USA). The content of α-tocopherol was determined using a diode array detector (VP series, Shimadzu, Japan), following European standards (EN–12822 [26]; EN–12823-1 [27]). The samples were treated with alkaline saponification with 60% potassium hydroxide, followed by extraction with diethylether. The standard used was α-tocopherol (purity ≥ 97.0%; Sigma-Aldrich, St. Louis, MO, USA).

### 2.6. Sensory Analysis

The samples of LL intended for the sensory analysis were taken from the right side of the carcass at 24 h after slaughter and vacuum-packed (Vac-star, 180 × 200 mm, 90 µm thick). The samples were then aged at 4 °C for 48 h, before being frozen at −20 °C for a storage period of one month until analysis. The meat samples were thawed inside the vacuum-package bags at 4 °C for 24 h prior to the sensory evaluation. Then, the muscles were removed from the bags and cut into 20 mm thick steaks. The steaks were prepared by grilling them on a double glass/ceramic plate grill (VCR 6L TL, Fiamma, Aveiro, Portugal) preheated to 200 °C, until an internal temperature of 70 °C was reached, as measured by a digital temperature probe inserted into each sample (AD14TH, Ama-Digit, Kreuzwertheim, Germany). Immediately after cooking, samples were cut into 2 cm^3^ cubes, whilst excluding the outer meat surface, and placed into sealed glass containers. Descriptive sensory analysis (DSA) was performed on these samples during one session, within individual booths and under controlled environmental conditions using a red light (ISO 8589 [28]). The panel consisted of ten trained individuals (ISO 8586 [29]). Six sets of two samples were presented to each judge during the session: one sample from the OG pigs and the other from the IG pigs. The meat samples from barrows and gilts were presented to the judges separately. To avoid possible effects of the order of presentation and first-order carryover effects, the sample sets were presented to the judges in different randomized orders. The judges were provided with water and bread to cleanse their palates between samples. The analysis was based on six descriptors (Table 3) using a 9-point scale.

### 2.7. Calculations

#### 2.7.1. Metabolizable Energy (ME)

The energy supply capacity of the diet was evaluated on the basis of the ME calculated according to accepted standards [30].

#### 2.7.2. Indexes Related to Human Health

The atherogenic (AI), thrombogenic (TI), and saturation (S/U) indexes were calculated according to Ulbricht and Southgate [31].

AI = (C12: 0 + 4 × C14: 0 + C16: 0)/[Σ MUFA^1^ + Σ (*n* − 6) + Σ (*n* − 3)]

TI = (C14: 0 + C16: 0 + C18: 0)/[0.5 × Σ MUFA + 0.5 × Σ (*n* − 6) + 3 × Σ (*n* − 3) + Σ (*n* − 3)/Σ (*n* − 6)]

S^3^/U^4^ = (C14:0 + C16:0 + C18:0)/Σ MUFA + Σ PUFA^2^

^1^ PUFA: polyunsaturated fatty acids, ^2^ MUFA: monounsaturated fatty acids, ^3^ S: saturated fatty acids, ^4^ U: unsaturated fatty acids.

#### 2.7.3. Statistical Analysis

Statistical analysis was performed using mixed linear models (procedure MIXED) in the SAS statistical package (SAS Institute, Cary, NC, USA). The model equation for sensory data included the fixed effect of diet and the random effect of judge. The model equation for the remaining measured parameters included the fixed effect of diet. The data in the tables are presented as least square means (LSM) and standard errors of the mean (SEM). The individual pig represented the experimental unit for weight gain, carcass characteristics, physical and chemical composition, and FA profile of LL. Only the average feed intake and feed conversion ratio was measured per group and thus was not analyzed statistically, providing only descriptive information. A significance level of 5% was used throughout the statistical evaluation of the data.

## 3. Results

### 3.1. Growth Performance and Carcass Characteristics of Pigs

The effect of the rearing system type on the growth performance was minor. Daily weight gain in the OG pigs tended to be numerically higher, but did not differ significantly in comparison with the IG pigs. Feed intake during the experiment was recorded for each group, and thus the feed conversion ratio for each group was calculated from this. The evaluation of carcass characteristics found no notable differences between the treatment groups (Table 4).

### 3.2. Physical and Chemical Traits of the Longissimus Lumborum Muscle of the Outdoor and Indoor Groups

The physical meat quality traits investigated were similar in both groups. The average values matched those deemed acceptable for pork by previous research and thus it could be assumed the meat did not have any quality issues. Meat chemical compositions were similar for pork from both raising systems, and did not significantly differ betwen the housing groups (Table 5).

### 3.3. Fatty Acid Profile of Longissimus Lumborum Muscle

The fatty acid profile showed some significant differences between the pigs from different housing types. Outdoor-raised pigs had lower concentrations of saturated fatty acids/SFA (palmitic and stearic), MUFA (eicosenoic), PUFA n6 (eicosatrienoic, arachidonic, and docosatetraenoic), a lower ratio of PUFA n − 6/PUFA n − 3, and S/U, and lower atherogenic and thrombogenic indexes than indoor-raised pigs.

### 3.4. Organoleptic Properties of Grilled Longissimus Lumborum Muscle

The judge scores for meat sensory attributes indicated significant differences between the housing groups. The IG pork obtained better evaluations for tenderness, juiciness and chewiness, but on the contrary, the OG pork obtained better ratings for overall acceptance.

## 4. Discussion

This experiment was performed with the aim of evaluating the effect of providing outdoor access to PBP on their growth, carcass characteristics, and *longissimus lumborum* muscle meat quality. From the conclusions of previous preliminary studies, where PBPs were compared with a commercial hybrid line in two different production systems, the outdoor rearing system proved to be a more suitable alternative system for this indigenous breed [32].The growth performance and carcass traits evaluated in this study were comparable to those results obtained from the PBP population in previous studies [33,34]. Regardless of the production system used, the results show that PBPs were able to utilize a local protein feed. This is a positive result in light of Europe’s efforts to reduce its dependence on imported protein feed [35]. The results of the growth performance in this study did not differ significantly among the housing systems tested; however, it is worth mentioning that small numerical differences between the groups were observed regarding daily weight gain of the OG pigs, which tended to be higher than that of the IG pigs. Similar results have been presented in other studies, where outdoor-raised pigs were found to grow faster [36,37], but other studies have also reported slower growth rates in outdoor pigs [38]. In the present study, no effects were observed on the carcass traits (weight of commercial cuts, lean meat content, and backfat thickness) in pigs allowed outdoor access. This result is comparable with a similar studies [39,40], except the characteristics of backfat thickness. However, comparison with other studies is problematic, because different characteristics have been observed for this indicator. Lower backfat thickness has been reported in several studies [40,41], but other studies have also reported higher backfat thicknesses for outdoor-raisied pigs [6]. The contradictory findings of these studies demonstrate that the effect of outdoor rearing may differ in pigs, depending on a variety of factors, as the systems used are not uniform. It is obvious that the benefits of outdoor-raising can thus only be attained under certain conditions. One such influencing factor is ambient temperature, which can significantly affect the growth rate and carcass characteristics of pigs. Temperatures above the upper critical level of the thermoneutral zone of pigs causes reduced feed intake, while temperatures below the lower critical level increases energy requirements, and leads to the deposition of subcutaneous fat [6]. Nevertheless, according to previous observations (unpublished data), there are factors other than temperature which may affect the intensity of pasture usage in outdoor-raised pigs, such as the configuration of the paddock and the attractiveness of the pasture fodder, which has been described not only in cattle [42] but also in pigs [43].

According to the physical and chemical traits of the LL muscle, there were no significant differences caused by the housing systems in which the pigs were raised in the present study. Both groups exhibited average pH_45_ and pH_24_ values considered normal for meat [44,45], as well as normal values for drip loss and water holding capacity. These results agree with other studies [12,39,46] in which no effect of raising pigs within outdoor systems was observed for the physical traits/quality of meat. The content of IMF did not significantly differ between the housing systems in the present study; although, the OG pigs tended to have numerically lower IMF levels, which may have been affected by the increased physical activity of free-ranging pigs [47]. Overall, there were no significant differences in the meat chemical composition between the housing systems in the present study, as similarly reported in other studies [3,37,48].

The fatty acid profile and indexes related to human health, shown in Table 6, were significantly affected by the housing systems used in the present study. The pork from OG had a significantly lower level of the SFAs: palmitic and stearic acid, and lower PUFAs (n6): docosatetraenoic, eicosatrienoic and arachidonic acid. The total SFA was only numerically lower in the pork from the OG, but the PUFA n − 6/PUFA n − 3 ratio, saturation, and atherogenic and thrombogenic indexes were significantly lower in the OG pigs in the current study. With regards to the general recommendation to reduce the risk of coronary heart disease in humans [23], these results are positive in the context of human health. Differences in fatty acid composition and indexes could be ascribed to the free access to pasture with a high PUFA n3 content for outdoor pigs; the positive influence of this on the fatty acid profile of the meat is well-known [6]. Furthermore, higher physical activity levels in free-ranging pigs is positively correlated with PUFA n3 levels in the meat, as discussed in the other studies [49,50].

The results regarding tocopherol in the current study did not meet initial expectations, as a higher level of tocopherol was expected in the pork from outdoor-raised pigs, in accordance with the known positive influence of grazing on tocopherol [6,51]. Although meat is not an important source of tocopherol for humans, interest in improving the oxidative status of meat and prolonging the storage life of meat is increasing. In the present study, the content of tocopherol did not differ significantly between the treatment groups; however, in the pork from OG pigs, there was a tendency for higher levels of tocopherol. The fact that the levels of tocopherol were non-significant could be ascribed to the varied levels of tocopherol in the pasture over the seasons, as a lower content of tocopherol and lower quality of forage may be found in late summer. Some studies have reported on the varied quality of pasture over the seasons [52], as well as the various tocopherol concentrations in the context of plant species and maturity stage [53].

Eating quality of the pork in the current study was significantly affected by the housing system used. The results of the sensory analysis of grilled meat (Table 7) demonstrated that the pork from the OG pigs received significantly poorer ratings for tenderness, juiciness, and chewiness, but better evaluations for overall acceptance. Comparisons of the effect of diet on the meat quality, between animals allowed to move freely and animals restricted in pens, lead to problems in interpreting data. The effect of feeding may further be confounded by the different levels of physical activity between the study groups [54]. The main differences for sensory eating quality in the present study were recorded for texture characteristics, where the IG group received more favorable assessments. Higher scores, particularly for juiciness and tenderness in the IG group, were probably associated with a slightly higher IMF.

There is no plausible explanation as to why the OG meat was assessed more favorably for overall acceptance than the IG meat in the present study, and thus this result may be due to differences in other sensory descriptors that were not the subject to this evaluation. Over 1000 volatile compounds responsible for differences in meat flavor have been identified [55], and some of them can be influenced by different dietary components. The diet-specific differences can thus only be partially explained by differences in the FA composition of muscle lipids [56]. Generally, a higher proportion of long, unsaturated FAs responsible for “grassy” and “game” flavors is found in meat from grazing animals, compared with animals fed a grain-based diet [54]. Unsaturated FAs are more prone to oxidation, and thus they may also contribute to the development of various “off-flavors” or “off-odors” in meat. This may therefore be the reason for the numerically lower appraisal of flavor and odor in the pork from pasture-fed OG animals. Overall, comparison with other relevant studies of similar experimental designs is rather difficult for the sensory eating quality of pork from different housing systems. The results published to date are inconsistent, and probably reflect the different conditions of finishing systems, which may affect the organoleptic properties of meat [3]. In current literature, studies with better scores for tenderness and juiciness for pork from indoor pigs compared with pork from outdoor pigs can be found [57,58]. On the other hand, studies also reported no significant differences in those same sensory attributes when comparing pork from indoor and outdoor rearing systems [3,59].

## 5. Conclusions

In the present study, the outdoor system produced meat with a more favorable fatty acid profile, particularly regarding indexes related to human health. The inclusion of outdoor-access in the raising of PBPs had no negative effects on their growth, carcass traits, and physical meat quality. Further research is however required to determine the optimal feeding regime and pasture management to optimize the meat quality of these pigs. Outdoor-access negatively affected some sensory characteristics of the pork from this system (tenderness, juiciness, chewiness). However, further sensory evaluation including more descriptors and a greater sample size should be performed, particularly considering the importance of higher-welfare systems and the consumer demand for locally produced high-welfare meat products.

## Figures and Tables

**Table 1:** Nutritional composition of the available pasture for the outdoor group.

Composition (g/kg) ^1^	Paddock 1	Paddock 2	Paddock 3
Dry matter	253.0	789.3	509.3
Ash	21.56	76.14	53.07
Crude protein	23.7	73.9	42.0
Ether extract	3.9	5.8	12.3
Crude fiber	72.9	304.7	142.0
**Fatty Acid** (%) ^2^			
Lauric C 12:0	0.06	0.15	0.29
Myristic C 14:0	0.29	0.70	0.63
Palmitic C16:0	14.07	16.05	17.60
Oleic C18:1n − 9	3.54	18.28	23.76
Linoleic C18:2n − 6	15.98	47.92	37.43
α-linolenic C18:3n − 3	59.91	8.11	13.97
Arachidonic C20:4n − 6	0.21	0,15	0.11
ME (MJ/kg) ^3^	1.7	3.2	3.2

^1,2^ Analyzed composition. ^1^ On an as-fed basis. ^2^ Percent of total fatty acids. ^3^ Metabolizable energy.

**Table 2:** Ingredients and nutritional composition of the experimental diet for both groups.

Ingredients (g/kg) ^1^	
Wheat	550
Pea seeds	120
White lupine seeds	100
Wheat bran	200
Vitamin/mineral premix ^2^	30
**Composition** (g/kg) ^1^	
Dry matter	870.4
Crude protein	145.7
Ether extract	30.0
Crude fiber	54.4
Lysine	7.1
Methionine	2.4
**Fatty Acid** (%) ^3^	
Lauric C 12:0	0.03
Myristic C 14:0	0.51
Palmitic C16:0	19.82
Oleic C18:1n − 9	26.92
Linoleic C18:2n − 6	35.73
α-linolenic C18:3n − 3	3.60
Arachidonic C20:4n − 6	0.10
ME ^4^ (MJ/kg)	12.4

^1,2^ Analyzed composition. ^1^ On an as-fed basis. ^2^ Premix of micro- and macrominerals, essential amino acids, and vitamin-Premix provided per kg of feed: retinol, 12.000 IU; cholecalciferol, 1980 IU; α-tocopherol, 180 mg; menadione, 3 mg; thiamine, 1.8 mg; riboflavin, 4.5 mg; pyridoxin, 3 mg; cobalamin, 0.03 mg; niacin, 24 mg; folacin, 0.45 mg; Ca pantothenate, 11.3 mg; choline Cl, 450 mg; Fe, 105 mg as FeSO_4_·H_2_O; Zn, 108 mg as ZnO; Mn, 93 mg as MnO; Cu, 9.3 mg as CuSO_4_·5H_2_O; I, 2.25 mg as Ca(IO3)_2_; Co, 0.45 mg as 2CoCO_3_·3Co(OH)_2_·H_2_O; Se, 0.39 mg as Na_2_SeO_3_; 6-phytase (EC 3.1.3.26), 750 FTU; Ca, 6 g; P, 0.9 g; Na, 1.5 g; Mg, 0.06 g; lysine, 3.09 g; methionine, 0.6 g; threonine, 0.6 g. ^3^ Percent of total fatty acid. ^4^ Metabolizable energy.

**Table 3:** Definition and scale of attributes used in the meat sensory analysis.

Attribute	Evaluation	Definition	Scale
Pork odor intensity	Before eating sample	The strength of aroma typical for cooked pork.	1 = very low 9 = very high
Tenderness	After first two or three chews	The force required to bite through the sample with molars.	1 = very low 9 = very high
Juiciness	After first three to five chews	The amount of moisture released by the sample.	1 = very low 9 = very high
Pork flavor intensity	After first five to ten chews	The presence of a flavor typical for cooked pork.	1 = very low 9 = very high
Chewiness	After at least 15 chews	The amount of residual tissue after most of the sample has been masticated.	1 = scarcely chewable 9 = easily chewable
Overall acceptance	After completion of evaluation	Preference of the judge among the evaluated samples.	1 = not acceptable 9 = highly acceptable

**Table 4:** Growth performance and carcass characteristics of Prestice Black-Pied pigs reared in different housing systems.

Item	Housing			
	Outdoor	Indoor	SEM	Significance
	*n* = 12	*n* = 12		*p*-Value
Initial body weight (kg)	21	23	2	NS
Final body weight (kg)	109	111	4	NS
Average weight gain (kg/day)	0.74	0.70	0.07	NS
Average feed intake ^1^ (kg/day)	2.70	2.68	−	−
Feed conversion ratio (FCR) ^1^	3.33	3.45	−	−
**Carcass characteristics** (kg)				
Right carcass side	42.4	43.2	1.6	NS
Ham ^2^	7.5	7.9	0.7	NS
Loin ^2^	4.4	4.3	0.5	NS
Shoulder ^2^	3.7	3.6	0.3	NS
Belly ^2^	8.4	8.8	0.7	NS
Back fat thickness (mm)	34.23	32.97	4.3	NS
Lean meat content (%)	53.83	53.75	2.13	NS

^1^ Data not analyzed statistically and excludes forage intake. ^2^ Cuts, with bones, from the right side of the carcasses. NS *p* > 0.05. SEM, standard error of the mean (*n* = 12 pigs per treatment).

**Table 5:** Physical and chemical traits of *longissimus lumborum* muscle of Prestice Black-Pied pigs reared in different housing systems.

Item	Housing			
	Outdoor	Indoor	SEM	Significance
	*n* = 12	*n* = 12		*p*-Value
Physical meat quality traits				
pH_45_	6.21	6.09	0.15	NS
pH_24_	5.58	5.55	0.17	NS
Drip loss %	3.01	3.02	0.71	NS
WHC ^1^ %	32.75	35.83	6.20	NS
Proximate chemical composition				
Dry matter (g/kg)	270	274	10	NS
Protein (g/kg)	227	227	6	NS
Lipids (g/kg)	25	28	10	NS
Cholesterol (g/kg)	482	503	34	NS
Hydroxyproline (g/kg)	0.47	0.47	0.04	NS
Tocopherol (mg/kg)	3.89	3.79	0.69	NS

^1^ WHC, water-holding capacity. NS *p* > 0.05. SEM, standard error of the mean (*n* = 12 pigs per treatment).

**Table 6:** Fatty acid profile (mg/100 g of muscle), and indexes related to human health, of the *longissimus lumborum* muscle of Prestice Black-Pied pigs reared in different housing systems.

Item	Housing			
	Outdoor	Indoor	SEM	Significance
	*n* = 12	*n* = 12		*p*-Value
SFA ^1^				
Lauric (C 12:0)	2.15	2.62	0.69	NS
Myristic (C 14:0)	33.24	41.38	11.16	NS
Pentadecanoic (C 15:0)	1.55	1.64	0.44	NS
Palmitic (C 16:0)	581.13	749.97	176.5	*
Margaric (C 17:0)	3.00	3.12	0.90	NS
Stearic (C 18:0)	240.92	323.87	89.32	*
Total SFA	869.36	1131.63	278.77	NS
MUFA ^2^				
Palmitoleic (C 16:1n − 7)	92.45	99.75	28.55	NS
Oleic (C 18:1n − 9)	994.62	1244.15	325.4	NS
(C 18:1n − 7)	90.05	98.78	27.92	NS
Eicosenoic (C 20:1n − 9)	13.19	17.46	4.32	*
Total MUFA	1192.14	1461.93	384.34	NS
PUFA ^3^				
Linoleic (C 18:2n − 6)	231.38	238.33	48.93	NS
α-linolenic (C 18:3n − 3)	55.50	37.46	19.82	NS
Eicosadienoic (C 20:2n − 6)	6.54	7.40	1.02	NS
Eicosatrienoic (C 20:3n − 6)	5.14	5.94	0.81	*
Arachidonic (C 20:4n − 6)	37.74	42.19	4.44	*
EPA ^4^ (C 20:5n − 3)	4.21	4.23	0.56	NS
Docosatetraenoic (C 22:4n − 6)	5.14	6.26	0.91	*
Docosapentaenoic (C 22:5n − 3)	4.21	4.23	1.09	NS
DHA ^5^ (C 22:6n − 3)	0.63	0.60	0.17	NS
Indexes				
Total PUFA n − 3	69.27	51.62	19.89	NS
Total PUFA n − 6	289.23	304.10	51.24	NS
PUFA n − 6/PUFA n − 3 ratio	4.47	6.32	1.55	**
S/U ^6^	0.71	0.79	0.05	**
Atherogenic index	0.46	0.51	0.02	**
Thrombogenic index	0.89	1.07	0.19	***

SEM, standard error of the mean (*n* = 12 pigs per treatment).^1^ SFA, saturated fatty acids; ^2^ MUFA, monounsaturated fatty acids; ^3^ PUFA, polyunsaturated fatty acids; ^4^ EPA, eicosapentaenoic acid; ^5^ DHA, docosahexaenoic acid; ^6^ S/U, saturated fatty acid/unsaturated fatty acid. NS *p* > 0.05, * *p* ≤ 0.05, ** *p* ≤ 0.01, *** *p* ≤ 0.001.

**Table 7:** Organoleptic properties of grilled *longissimus lumborum* muscle.

Item	Housing			
	Outdoor	Indoor	SEM	Significance
	*LSM*	*LSM*		*p*-Value
Pork odor intensity	4.1	4.0	0.3	NS
Tenderness	4.2	5.1	0.2	***
Juiciness	4.6	6.0	0.2	***
Pork flavor intensity	3.9	4.3	0.2	NS
Chewiness	4.3	5.4	0.2	***
Overall acceptance	5.3	4.2	0.2	***

SEM, standard error of the mean (*n* = 12 pigs per treatment). NS *p* > 0.05, *** *p* ≤ 0.001.
